# A Rare Case of Sonographically Detected Silent Rupture of a Gravid Uterus With Expulsion of a Complete Fetus and an Intact Amniotic Sac

**DOI:** 10.7759/cureus.89979

**Published:** 2025-08-13

**Authors:** Anupam Rajoria, Mukesh Mittal, Gunjan Aggarwal

**Affiliations:** 1 Department of Radiodiagnosis, SMS Medical College, Jaipur, IND; 2 Department of Radiodiagnosis and IR, All India Institute of Medical Sciences, New Delhi, New Delhi, IND

**Keywords:** antenatal usg, intact amniotic sac, placental herniation, previous c section, silent uterine rupture

## Abstract

Uterine rupture is an infrequent complication that can threaten the survival of both mother and growing fetus. Prelabor silent uterine rupture with expulsion of an intact amniotic sac into the abdominal cavity is very rare. We report such a case of a 23-year-old pregnant woman who presented for a routine antenatal ultrasound scan in her third trimester. Her previous scan, done elsewhere at 23 weeks of gestation, had shown a live intrauterine fetus. Our antenatal USG scan showed a full-thickness defect in the lower uterine wall with placental herniation plugging the defect and the rest of the products of conception (i.e., a nonviable fetus with an intact amniotic sac) in the abdominal cavity. The patient underwent surgery, which confirmed our findings. Diagnosis of the entity is made intraoperatively unless a thorough antenatal USG examination is done. Early diagnosis and timely management are critical for preserving life and fertility.

## Introduction

Uterine rupture in pregnancy may occur before or during delivery. Previous scarring in the uterus predisposes to this condition. While uterine rupture is seen in one in 5,000 to one in 7,000 pregnancies [1], clinically silent uterine rupture with expulsion of a complete fetus and intact amniotic sac is extremely rare, and only around 30 cases have been reported worldwide [2]. It poses significant risks to the mother and fetus. The rupture of a scarred uterus can occur spontaneously or as a result of trauma, often due to obstetric procedures [3]. Diagnosis can be challenging and may be made only intraoperatively unless confirmed on ultrasonography (USG) scan beforehand [4].

## Case presentation

A 23-year-old pregnant female in her third trimester came for antenatal evaluation in our tertiary healthcare center. She had an obstetric history of two prior pregnancies, which had both been delivered by lower-segment caesarean section (LSCS), the first of which had been done given breech presentation and the second for prior caesarean section. Both previous pregnancies had been uneventful, and her surviving child was healthy (one lost in infancy because of gastrointestinal infection and resulting sepsis). Her last conception had been two years back. Her last antenatal ultrasound scan had been done elsewhere two months back and had shown a live intrauterine fetus of age 23 weeks.

Though she complained of previous and ongoing episodes of vague lower abdominal pain during this pregnancy, she had not consulted any healthcare center for the same. There was no complaint of leaking or bleeding per vagina.

Her per abdominal examination demonstrated mild tenderness, fundal height consistent with gestational age, and absent fetal movements and heart sounds. Her vital parameters were stable. Mild pallor was present.

**Figure 1:**
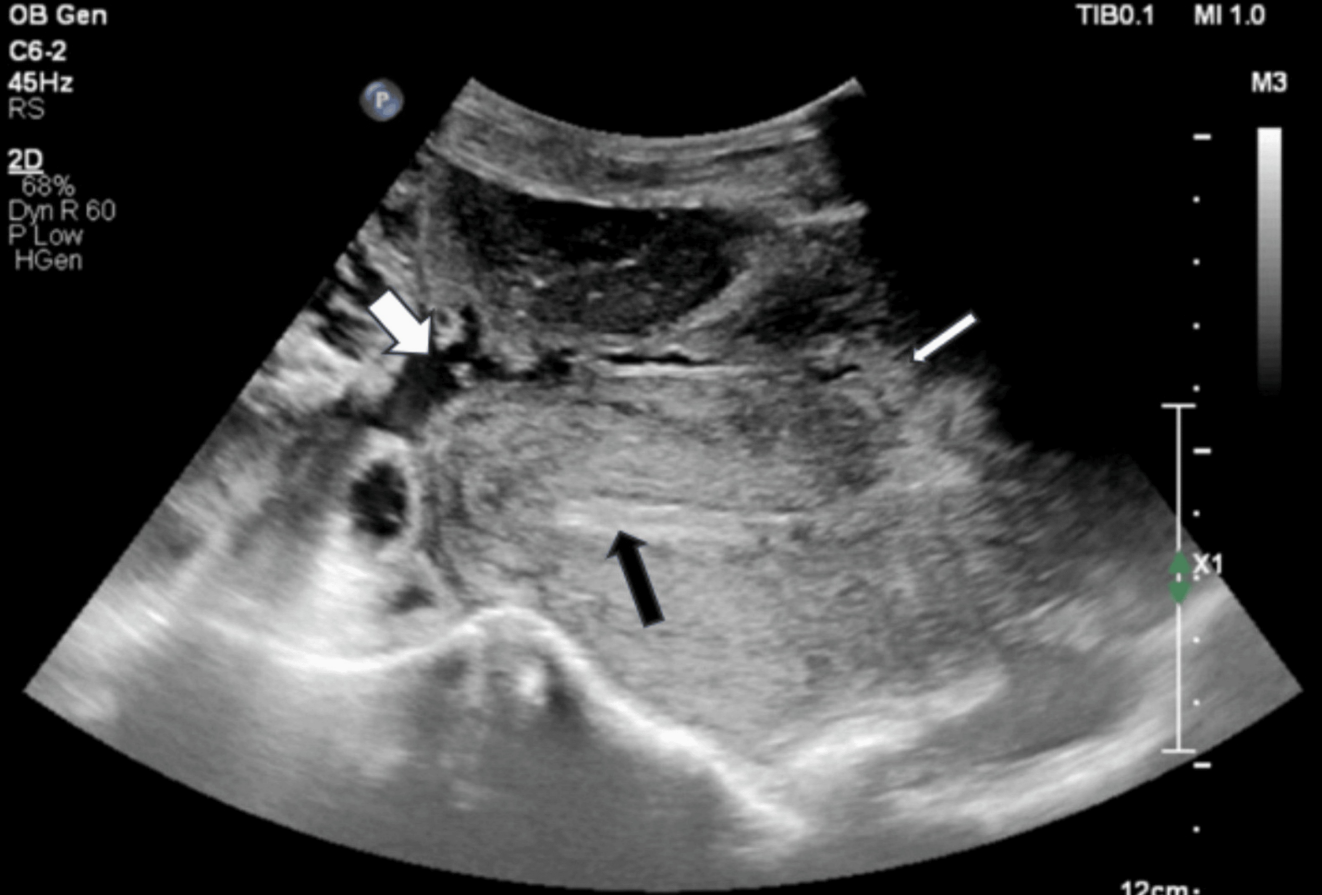
Sagittal USG image. Sagittal USG image showing a bulky uterus, empty endometrial cavity (black arrow), closed os, and placenta herniating through the caesarean scar site defect in the lower anterior wall (thin white arrow). Intra-abdominal extrauterine fetal parts are partially visualized (thick white arrow).

An ultrasound scan was performed to assess fetal well-being. The USG scan revealed an empty uterine cavity. At the presumed site of her previous LSCS, there was a discontinuity in the entire thickness of the lower anterior uterine wall, including the serosa (Figure 1).

**Figure 2:**
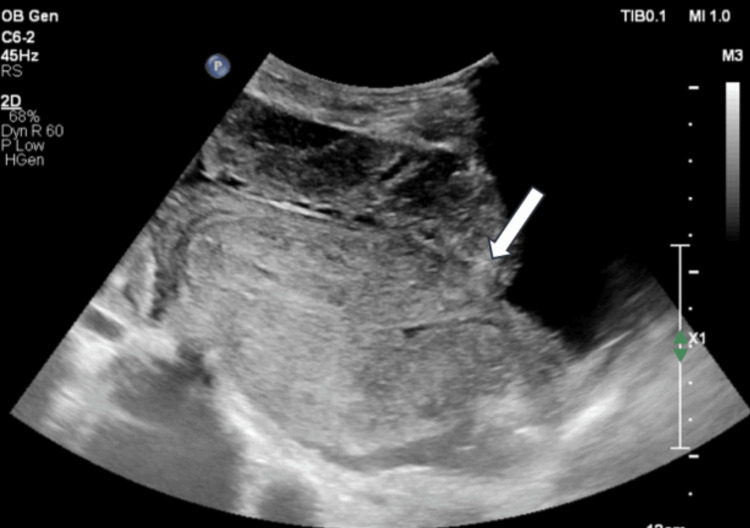
Sagittal USG image. Sagittal USG image focusing on placenta herniating (arrow) from ruptured uterus and lying apposed to the urinary bladder and anterior abdominal wall. Free fluid in rectouterine space is present.

Through the discontinuity, the placenta appeared to be herniating upward from the uterus (Figure 2) and was in close relation to the anterior abdominal wall.

**Figure 3:**
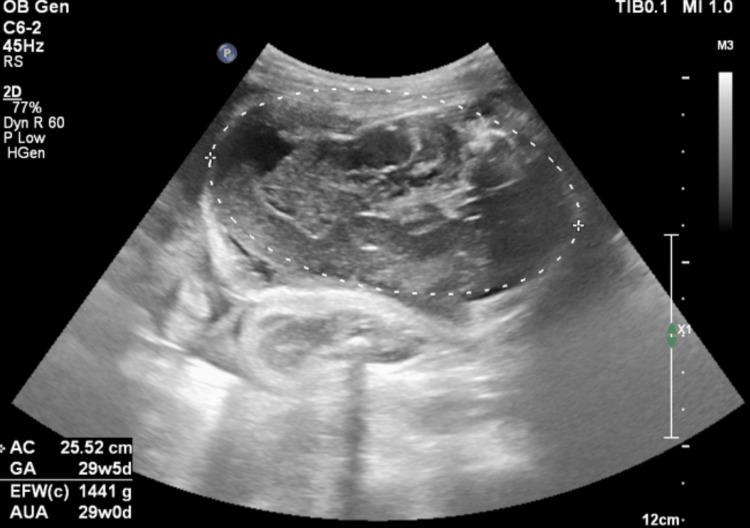
Axial USG of extrauterine fetal hydrops. USG image showing axial section of extrauterine compressed fetal abdomen in abdominal cavity showing hydrops.

The fetus was located in the abdominal cavity. Its amniotic sac was preserved. There was no fetal cardiac activity and no fetal movements. Fetal biometry using femur length showed a gestational age of around 32 weeks. Fetal anasarca and distorted abdominal circumference were also present (Figure 3). The presence of the Spalding sign, or overlapping of fetal cranial bones, indicated fetal demise that had occurred within the previous week or more. There was reduced liquor.

The rest of the maternal abdomen was examined and showed minimal anechoic free fluid in the abdominal cavity.

Based on these findings, we diagnosed uterine rupture with placental herniation and intact amniotic sac in the abdominal cavity with fetal demise. The patient was taken up for laparotomy. Intraoperatively, the placenta was found lying directly beneath the abdominal wall, without evidence of adherence to the wall. The gestational sac was intact. Uterine repair was done, given the patient’s young age and desire for further conception. Patient recovery was uneventful.

## Discussion

Silent uterine rupture can be spontaneous or caused by external injury, connective tissue disorders, rupture of a previous LSCS scar, uterine malformation, or placental abruption, among others [5]. Our case had undergone two prior LSCS, and uterine rupture occurred at the scar site. The patient had a previous ultrasound scan report that showed a live intrauterine fetus at 23 weeks of gestation. This suggests that the silent uterine rupture occurred in the third trimester. Because the patient’s intermittent pain was ignored and there were no severe symptoms, it cannot be confirmed exactly when the rupture occurred. Fetal demise had occurred at least one week before our scan; however, in the absence of a confirmed chronology, it is plausible that it might have survived for some weeks outside the uterus.

With myometrial contraction and possible tamponade by the extruded fetus, the rupture can remain clinically silent. USG examination may miss the diagnosis if the contracted uterus is misinterpreted as placenta previa, especially in the presence of vaginal bleeding [6]. Hence, a thorough evaluation is mandatory. It is advisable for all patients in the third trimester that lower abdominal pain or bleeding should not be neglected, and healthcare should be sought at the earliest for favorable outcomes.

Cases of silent uterine rupture are difficult to diagnose because of their unclear and diverse symptoms, which can overlap with complaints of other common abdominal and gynecological conditions. Lower abdominal pain and vaginal bleeding may occur, but additional gastrointestinal symptoms such as nausea, vomiting, diarrhea, and constipation can further complicate the diagnosis, making it harder to distinguish from other issues such as gastrointestinal disorders or ectopic pregnancies [7]. In this case, the patient complained of vague off-and-on abdominal pain and only disclosed this with leading questions.

Prediction of uterine rupture is practically impossible. A targeted ultrasound examination of the uterine wall at the site of a previous uterotomy can be considered; however, this method also has limitations. There are no clear-cut threshold values for uterine wall thickness that can reliably predict uterine rupture. In cases where the findings are unclear and the status of the mother and fetus is stable, performing an MRI can be beneficial [4]. However, in our case, because fetal demise had already occurred, an MRI was not performed, and the patient was directly taken to the operating theater after the ultrasound scan.

There have been rare instances where uterine rupture has led to the successful delivery of a live, healthy neonate. Woo et al. reported a rare case of silent, spontaneous uterine rupture with the successful delivery of a healthy male infant, highlighting the unusual possibility of a favorable outcome in such critical situations [8].

As far as maternal outcomes are concerned, early surgical intervention is crucial for improving the chances of recovery and minimizing complications. Without a clear medical consensus, it is prudent to perform surgical exploration early. Laparotomy provides rapid and effective hemostasis, which is a significant advantage over laparoscopic surgery and may be considered particularly in the early stages of pregnancy [9]. Conservative management is possible only in asymptomatic cases with good maternal and fetal health, accompanied by intensive monitoring of both. The patient and medical staff must be prepared to address sudden complications, particularly those involving life-threatening intra-abdominal bleeding, and the patient should be counseled on the risk of recurrent rupture. The decision to preserve the uterus depends on the clinical circumstances, with factors such as the size of the defect and the placement of the placenta playing a critical role.

## Conclusions

Silent rupture of the gravid uterus is uncommon and potentially life-threatening for both mother and fetus. Clinical detection is often impossible prior to surgery; however, imaging serves as an excellent tool to guide management. We illustrate the vital role of timely and thorough antenatal ultrasound scanning in the diagnosis of this entity. Our case was unique, as complete expulsion of an intact amniotic sac is rarely reported in world literature. The case highlights the importance of timely imaging and early surgical intervention to minimize maternal mortality.
